# Tuning Liposome Stability in Biological Environments and Intracellular Drug Release Kinetics

**DOI:** 10.3390/biom13010059

**Published:** 2022-12-27

**Authors:** Keni Yang, Karolina Tran, Anna Salvati

**Affiliations:** Department of Nanomedicine and Drug Targeting, Groningen Research Institute of Pharmacy, University of Groningen, A. Deusinglaan 1, 9713 AV Groningen, The Netherlands

**Keywords:** liposome, liposome stability, biological environment, release kinetics, intracellular drug release

## Abstract

Ideal drug carriers should be stable in biological environments but eventually release their drug load once inside the targeted cells. These two aspects can be in contrast with each other, thus they need to be carefully tuned in order to achieve the desired properties for specific applications. Quantifying drug release profiles in biological environments or inside cells can be highly challenging, and standard methods to determine drug release kinetics in many cases cannot be applied to complex biological environments or cells. Within this context, the present work combined kinetic studies by flow cytometry with aging experiments in biological fluids and size-exclusion chromatography to determine drug release profiles in biological environments and inside cells. To this purpose, anionic and zwitterionic liposomes were used as model nanomedicines. By changing lipid composition, liposome stability in serum and intracellular release kinetics could be tuned and formulations with very different properties could be obtained. The methods presented can be used to characterize liposome release profiles in complex biological media, as well as inside cells. In this way, liposome composition can be tuned in order to achieve formulations with optimal balance between stability and release kinetics for specific applications.

## 1. Introduction

Liposomes are one of the most clinically established therapeutic delivery platforms in nanomedicine [1,2,3]. Since the introduction in 1995 of the first “nanotherapuetic”, Doxil, in the market, nowadays several liposomal drugs are used routinely in the clinic [2,4,5,6,7]. Taking advantage of the capacity of phospholipids to self-assemble, liposomes with varied charge and surface properties can be prepared by simply changing the lipid composition, and tuning liposome composition also allows changing bilayer properties in order to affect the biodistribution and pharmacokinetics of the loaded cargo [8,9]. 

It is generally believed that drug release from liposomes following endocytosis occurs either through the fusion between liposomes and the endosomal membrane [10,11,12,13] or by the diffusion of the encapsulated molecule across lipid bilayers [14,15,16]. In all cases, the unloading of the encapsulated cargo from liposomes following endocytosis by the target cell is an important and usually rate-limiting step [17,18,19]. For instance, Seynhaeve et al. have shown that free doxorubicin is more effective in killing cells than a liposomal formulation, such as Doxil, since the free drug enters cells rapidly by diffusion, accumulates in the nucleus and kills cells readily, while in order to reach the nucleus, the drug entrapped in liposomes first needs to be released [20]. Stable liposomal formulations are usually developed in order to ensure a long circulation time in vivo, but liposome stability may limit drug release once the liposome arrives at the diseased sites and reaches the targeted cells [20]. On the other hand, the early release of the loaded cargo from liposomes may lead to increased toxicity and side effects and limit therapeutic efficiency [21]. Therefore, to maximize the benefits of liposomal formulations, an optimal balance between minimal drug leakage in blood and efficient drug release in the targeted cells has to be established [18,22]. More importantly, in order to achieve this optimal balance, robust in vitro drug release testing methods are required to be able to determine release kinetics in complex biological fluids, such as blood, as well as inside cells [23].

Despite the importance of determining such parameters, no standardized methods are available yet for this purpose [23,24]. Dialysis and centrifugation are commonly used for quantifying in vitro drug release, typically in simple buffer solutions [6,24,25,26]: the released drug is separated from the drug-loaded liposomes by either a dialysis membrane or by ultracentrifugation and then quantified using methods such as UV/fluorescence spectroscopy or HPLC. However, these separation methods cannot be used to determine release kinetics inside cells and at the same time are likely to affect the rate of drug release. Washington et al. for instance argued that, in dialysis, the drug release rate is affected by the high concentration gradient between the large release medium in the donor compartment and the bulk phase in the receptor compartment [27,28]. Additionally, Moreno-Bautista et al. showed that the quantification of drug release by dialysis is not accurate when the actual release rate of drugs from liposomes is higher than the rate of diffusion from the dialysis membrane [29]. Similarly, for centrifugation-based methods, the high centrifugal force applied may increase drug release or even damage the liposomes [30]. Other testing methods have been developed to try to overcome some of these limits, such as the application of two-stage reverse dialysis by Xu et al. [31]. However, including the effects of serum protein binding and corona formation on liposome stability [24] and determining release kinetics inside cells following uptake and intracellular trafficking poses ulterior challenges in conducting and interpreting in vitro release studies of liposomal formulations. 

Within this context, in the present study, flow cytometry analysis, imaging by fluorescence microscopy, aging experiments in biological media and size-exclusion chromatography have been combined in order to determine the release behavior of liposomes upon exposure to complex biological fluids and inside cells. An anionic liposome (DOPG liposome) and a zwitterionic liposome (DOPC liposome) showing very different physicochemical properties and cellular uptake behavior were used as liposome models, and sulforhodamine B (SRB), a membrane impermeable fluorophore, was used to mimic hydrophilic drugs entrapped in the inner aqueous volume of liposomes and to quantify liposome release behavior [32]. After exposure to liposomes for different times, intracellular release kinetics were obtained by quantifying how the fluorescence of the internalized SRB changed over time after removal of the extracellular liposome dispersion. In order to study the stability of liposomes in complex biological fluids such as serum, aging of liposome dispersions in serum and size-exclusion chromatography were combined in order to quantify eventual drug leakage following the interaction with serum and corona formation. Overall, the methods applied allowed us to determine release kinetics in serum and inside cells and to compare the different liposome formulations. The same approaches can be used to characterize these crucial properties for different liposomes, as well as for other drug carriers.

## 2. Materials and Methods

### 2.1. Liposome Preparation and Characterization

Lipids including 1,2-dioleoyl-sn-glycero-3-phosphocholine (DOPC), 1,2-dioleoyl-sn-glycero-3-phospho-(1’-rac-glycerol) (DOPG) and cholesterol were purchased from Avanti Polar Lipids, Inc., Alabaster, AL, USA. DOPC liposomes composed of DOPC and cholesterol at a molar ratio of 2:1 and DOPG liposomes composed of DOPG and cholesterol at a 2:1 molar ratio were both prepared by thin-film hydration followed by freeze–thaw cycles and extrusion. Briefly, 10 mg lipid mixture was dissolved in chloroform, and the organic solvent was evaporated with dry nitrogen for 30 min, followed by further removal under vacuum overnight. Lipid films were rehydrated with 1 mL 25 mM sulforhodamine B (SRB) in PBS followed by 8 freeze–thaw cycles using liquid nitrogen and a 37 °C water bath and extrusion 21 times through a 0.1 µm polycarbonate membrane using an Avanti Mini-Extruder (Avanti Polar Lipids) in order to obtain small unilamellar liposomes. Zeba Spin Desalting Columns (7K MWCO, from Thermo Fisher Scientific, Waltham, MS, USA) were used to remove excess free SRB, and the obtained liposomes were stored at 4 °C and used up to 1 month after preparation.

The concentration of DOPC or DOPG after extrusion and free dye removal was quantified via the Stewart assay, which allows the quantification of the concentration of phospholipids [33]. For this, a ferrothiocyanate reagent was first prepared by dissolving 27.03 mg ferric chloride hexahydrate (Sigma Aldrich, Darmstadt, Germany) and 30.4 mg ammonium thiocyanate (Sigma Aldrich) in 1 mL Milli-Q water. A total of 20 µL liposome stock was mixed with 1 mL chloroform and 1 mL ferrothiocyanate reagent, vortexed for 1 min and centrifuged for 10 min at 300 g. The chloroform phase was then transferred to a quartz cuvette, and the absorbance at 470 nm was measured using a Unicam UV500 Spectrophotometer (Unicam Instruments, Cambridge, UK). Solutions of DOPC or DOPG lipids at known concentration in the range from 0 mg/mL to 0.1 mg/mL were measured using the same method and the results used to build a calibration curve. This was used to quantify the final phospholipid concentration, and from this the total lipid concentration was calculated by assuming that the molar ratio between the phospholipid and cholesterol remained the same after extrusion.

In order to compare the fluorescence of SRB when encapsulated in the liposomes and after release from the liposomes, 62 µg/mL DOPC and DOPG liposomes were dispersed respectively in PBS and 1% triton (*v/v*) (Sigma Aldrich), and their fluorescence was measured using a ThermoMAX microplate reader (Molecular Devices, LLC, San Jose, CA, USA), with excitation at 550 nm and emission at 600 nm (negligible fluorescence was detected with these settings for triton solutions at this concentration). Addition of 0.5% (*v/v*)) triton has been shown to completely disassemble liposomes in dispersions at 2 mg/mL lipid [34,35]. Thus, here 1% triton was used to ensure a complete lysis of DOPC and DOPG liposomes. 

Size distributions and zeta potential of liposomes were measured using a Malvern Zetasizer Nano ZS (Malvern Instruments Ltd., Malvern, UK). Briefly, 62 µg/mL liposomes were dispersed in PBS or the complete MEM cell culture medium supplemented with 10% *v/v* FBS (cMEM), and the suspension was loaded in a 40 µL microcuvette (Malvern Panalytical, Malvern, UK) for size measurement immediately after preparation. The measurement was run with an automatic measurement duration at 20 °C. For zeta potential, suspensions were loaded on a disposable folded capillary cell (Malvern Panalytical), and samples were measured at 20 °C with automatic settings and analyzed using a monomodal model for high conductivity media. For each sample, three measurements were performed, and the results were the average and standard deviations over the 3 replicate measurements. 

### 2.2. Cell Culture

HeLa cells (ATCC CCL-2) were grown in a complete cell culture medium (cMEM) consisting of MEM (Gibco Thermo Fisher Scientific, Carlsbad, CA, USA) supplemented with 10% *v/v* fetal bovine serum (FBS, Gibco Thermo Fisher Scientific) in a humidified atmosphere with 5% CO_2_ at 37 °C. Cells were defrosted and kept in culture up to a maximum of 20 passages. Cells were tested monthly against mycoplasma to exclude contamination. 

### 2.3. Flow Cytometry 

The cellular uptake of liposomes was studied first by flow cytometry analysis. HeLa cells were seeded in a 24-well plate with a density of 5 × 10^4^ cells per well and cultured in cMEM for 24 h. Cells were then exposed to roughly 62 µg/mL of DOPC liposome or DOPG liposome dispersed in cMEM. After exposure, cells were collected at different time points by washing with cMEM once and PBS twice to remove excess liposomes and detached from the plate by incubation with 0.05% trypsin–EDTA for 5 min at room temperature. Cells were centrifuged at 300 g for 5 min, resuspended in 100 µL PBS and measured immediately using a BD FACSArray (BD Biosciences, San Jose, CA, USA) with a 532 nm laser. Dead cells and cell doublets were excluded by setting the gates in the forward scattering and side scattering plots, and for each sample, at least 20,000 cells were acquired. For each condition, two independent replicate samples were measured. Data were analyzed using FlowJo (FlowJo, LLC, Ashland, OR, USA) and exported as the average of the median cell fluorescence intensity and standard deviation over duplicates. Each experiment was repeated at least 2 times to confirm reproducibility. 

For uptake studies in serum free medium (sfMEM), prior to exposure to liposomes, cells were washed with sfMEM 3 times and incubated in sfMEM for 30 min. Then, cells were exposed to 62 µg/mL liposomes dispersed in sfMEM. The uptake of 5 µM free sulforhodamine B (SRB) (which roughly corresponds to the final concentration of SRB encapsulated in 62 µg/mL liposome) dissolved in cMEM or sfMEM was also measured in the same way as a control.

In order to study the intracellular release and eventual export of the liposomes and SRB, pulse-chase experiments were performed both in cMEM and sfMEM. Cells, seeded as described above, were incubated with 62 µg/mL DOPC or DOPG liposome dispersed in cMEM or in sfMEM for different “pulse” times, followed by the removal of the extracellular liposome dispersion and 3 washes with cMEM or sfMEM to remove the excess liposomes. Cells were then cultured in cMEM or in sfMEM in a humidified atmosphere with 5% CO_2_ at 37 °C and collected at different “chase” times for flow cytometry measurement as described above. As additional controls, similar pulse-chase experiments were performed on cells exposed to 5 µM free SRB or (given the very low uptake of free SRB) 10 times higher concentration (50 µM) in order to allow a higher signal inside cells to be detected. 

In order to study the stability of liposomes in biological fluids and upon corona formation, 62 µg/mL liposome dispersions in cMEM and sfMEM were prepared and kept in Eppendorf tubes with caps closed for increasing times in a humidified atmosphere with 5% CO_2_ at 37 °C as during exposure to cells. Then, HeLa cells seeded as described above were exposed for 2 h to dispersions aged for increasing times, and cell fluorescence was measured by flow cytometry. 

In order to study uptake and release in energy-depleted cells, cells were exposed to 5 mg/mL NaN_3_ (Merck Millipore, Darmstadt, Germany) for 30 min, followed by exposure to 62 µg/mL liposomes or 5 µM SRB in cMEM with 5 mg/mL NaN_3_. For pulse-chase experiments in energy-depleted cells, after exposure to liposomes or 5 µM or 50 µM free SRB for 2 h in standard conditions, cells were washed 3 times with cMEM to remove the excess liposome or free dye, then cultured for increasing “chase” times with either cMEM or cMEM supplemented with 5 mg/mL NaN_3_, followed by flow cytometry measurements. In order to measure the uptake of aged liposome dispersions in energy-depleted cells, the aged liposome dispersions prepared as described above were collected at different times, mixed with 5 mg/mL NaN_3_ and exposed for 2 h to cells pre-treated with 5 mg/mL NaN_3_ for 30 min. 

We note that many different batches of liposomes were used to generate the data reported, thus a direct comparison of fluorescence intensities in different panels is not possible because encapsulated SRB amounts differed slightly among batches. However, the general behavior and effects reported were the same in all experiments.

### 2.4. Fluorescence Imaging

Fluorescence microscopy was used to track liposome release inside cells. Briefly, 1.5 × 10^5^ cells were seeded in a 35 mm dish with a 170 µm thick glass bottom. After 24 h, cells were exposed to 62 µg/mL DOPG liposome in cMEM for 10 min or 120 min. Then, cells were washed 3 times with cMEM to remove excess liposomes, stained with 1 µg/mL Hoechst 33342 (Thermo Fisher Scientific) in cMEM for 5 min for nucleus visualization, washed with cMEM once again and then kept in cMEM with no phenol red (Thermo Fisher Scientific) for imaging experiments. The microscopy dish was then immediately transferred on a DeltaVision Elite microscope (GE Healthcare Life Science, Chicago, IL, USA), with a humidified chamber and 5% CO_2_ at 37 °C. After 40 min chase, images were acquired using a DAPI filter for Hoechst excitation and a TRITC filter for liposomes. In order to avoid bleaching, images were taken every 20 min for up to 200 min (11 images in total).

ImageJ software (http://www.fiji.sc) was used for image processing, and brightness and contrast were adjusted using the same setting for all images and samples in order to allow better visualization. To quantify the cell fluorescence intensity, individual cells that were fully included in the field of view were selected manually using the brightfield image as a reference to define their borders. Then for each time, the mean intensity of the selected area in each channel was obtained and the average mean intensity and standard deviation over two single cells in the same frame calculated. 

### 2.5. Size-Exclusion Chromatography

In order to study the stability of liposomes after exposure to a medium with serum and corona formation, size-exclusion chromatography was used to separate and quantify eventual free SRB leaking from the liposomes. Briefly, 1 mL samples of 62 µg/mL DOPG or DOPC liposomes in phenol-red free cMEM were incubated for increasing times in Eppendorf tubes with caps closed in a humidified atmosphere with 5% CO_2_ at 37 °C as during exposure to cells. Then, after different aging times, the dispersions were recovered and loaded on a Sepharose CL-4B (Sigma-Aldrich) column (15 × 1.5 cm) pre-balanced with PBS. Fractions of 0.5 mL eluent were collected up to a total volume of 15 mL (30 fractions). and 50 µL of each fraction was mixed with 50 µL 1% triton (*v/v*) to destroy the eventual liposomes and to fully release the encapsulated SRB. For each collected fraction, the fluorescence of the mixture at 600 nm was measured after excitation at 550 nm using a ThermoMAX microplate reader (Molecular Devices). In this way, elution profiles were determined, and the peaks corresponding to the elution of SRB encapsulated in the liposomes and eventual free SRB were obtained. The areas of the 2 peaks were then calculated and used to quantify the fraction of encapsulated and free SRB. For comparison, samples of 1 mL 62 µg/mL DOPG or DOPC liposome dispersed in PBS were also loaded on the column and fractions were collected and measured in the same way as described above.

## 3. Results and Discussion

### 3.1. Liposome Preparation and Characterization

**Figure 1:**
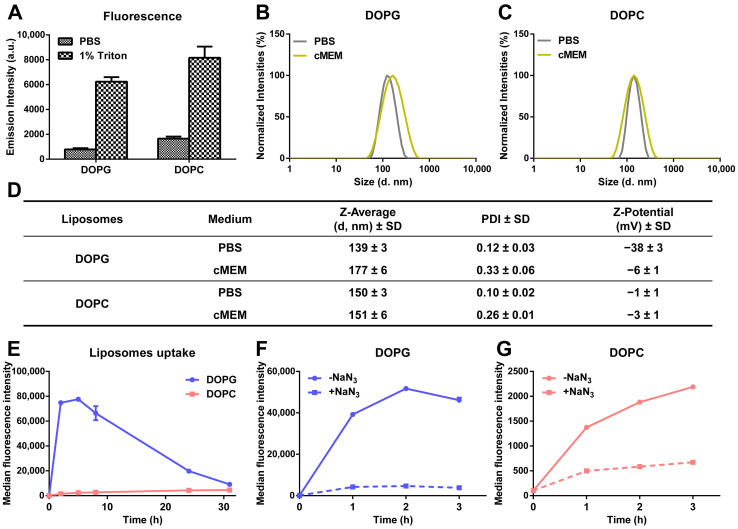
Characterization of the physicochemical properties and cellular uptake behavior of liposomes. (**A**) SRB fluorescence of liposomes dispersed in PBS and 1% triton. A total of 62 µg/mL liposomes was dispersed in PBS and 1% triton, and SRB fluorescence was measured immediately. The mean and standard deviation of the results obtained on 3 replicate samples are reported. Higher fluorescence was detected after dispersion in triton, indicating that the fluorescence of SRB was partially quenched inside liposomes. (**B**,**C**) Size distribution by intensity of DOPG (**B**) and DOPC (**C**) liposomes in different media. A total of 62 µg/mL liposomes was dispersed in PBS and cell culture medium supplemented with 10% FBS (cMEM) and their size distribution measured by DLS as described in Methods. The narrow distributions and low polydispersity confirmed the homogenous properties of liposomes after preparation by extrusion and when introduced in cMEM liposomes remained stable. (**D**) Z-average hydrodynamic diameter (d, nm) and polydispersity index (PDI) extracted by cumulant analysis of DLS data and zeta potential of liposomes dispersions (62 µg/mL) in PBS and cMEM. (**E**) Uptake kinetics of liposomes in cMEM. HeLa cells were exposed to 62 µg/mL DOPG or DOPC liposomes in cMEM and cells were collected for flow cytometry measurement after different exposure times as described in Methods. The results are the mean and standard deviation over 2 replicates of the median cell fluorescence intensity. (**F**,**G**) Uptake of DOPG (**F**) and DOPC (**G**) liposomes in energy-depleted cells. Briefly, Hela cells were pre-incubated with 5 mg/mL sodium azide (NaN_3_) for 30 min to deplete cell energy, then exposed to 62 µg/mL liposomes in cMEM in the presence of NaN_3_, followed by cell fluorescence measurement by flow cytometry as described in Methods. Uptake in standard conditions was also measured for comparison (-NaN_3_). Exposure to NaN_3_ strongly reduced uptake, indicating that both liposomes were internalized by cells via energy-dependent pathways.

Drug release kinetics were investigated for anionic liposomes and zwitterionic liposomes composed, respectively, of either 1,2-dioleoyl-sn-glycero-3-phospho-(1′-rac-glycerol) (DOPG) and cholesterol or 1,2-dioleoyl-sn-glycero-3-phosphocholine (DOPC) and cholesterol at a 2:1 molar ratio [32]. Liposomes were labelled with sulforhodamine B (SRB) to mimic hydrophilic drugs and quantify their release behavior using fluorescence-based methods. Importantly, disassembly of the liposomes in 1% triton showed increase in SRB fluorescence intensity, indicating that, for both formulations, the fluorescence of SRB trapped in the liposome lumen was partially quenched because of the high encapsulation efficiency (Figure 1A). Dynamic light scattering (DLS) and ζ-potential measurements in PBS or cell culture medium supplemented with 10% fetal bovine serum (cMEM) confirmed that the liposomes were well dispersed and had a very low polydispersity index (PDI) and that stable dispersions were also obtained in cMEM (Figure 1B–D). We previously showed that the dispersions in cell culture medium supplemented with serum remained stable also after 24 h incubation in biological conditions (5% CO_2_ at 37 °C), as used for experiments with cells [32]. The zeta potentials in PBS reflected the different lipid composition, but as commonly observed, once introduced in cMEM, they converged on similar values (around −5 mV) upon interaction with serum and corona formation on their surface (Figure 1D). 

When added to cells, as previously observed, the uptake of DOPC liposomes increased slowly with increasing exposure time, while DOPG liposomes showed much higher uptake in the first few hours, followed by a gradual decrease in average cell fluorescence, converging on values comparable to DOPC after around 30 h of exposure (Figure 1E) [32]. We previously showed that different mechanisms are involved in the uptake of the two liposomes [36]. However, in the presence of the metabolic inhibitor sodium azide (NaN_3_) to deplete cell energy, the uptake of both liposomes was strongly reduced (around 90 and 70% uptake reduction, respectively, for DOPG and DOPC liposomes) (Figure 1F,G), confirming that both liposomes were internalized as intact nanoparticles following an energy-dependent endocytic process [32]. The residual fluorescence in energy-depleted cells is likely due to the presence of some liposomes adhering outside of the cells [37], whose contribution in proportion is slightly higher in the case of DOPC liposomes, due to their much lower uptake efficiency. Fluorescence imaging in previous studies with the same liposomes confirmed the different uptake efficiency, as observed by flow cytometry, and showed that both liposomes were internalized by cells and trafficked to the lysosomes, as indeed expected following active uptake via endocytosis [32]. As a control, for comparison, additional studies were performed to determine the uptake of the same amount of free SRB. The results showed that free SRB uptake was rather low, reaching levels comparable to DOPC liposomes (Figure S1A) and, importantly, exposure to NaN_3_ had only minor effects, leading to around 30% uptake reduction (Figure S1B). These results suggested that the fluorescence detected in cells incubated with liposomes was mainly due to the active uptake of labelled liposomes rather than free SRB leaking from them.

### 3.2. Intracellular Release of Liposomes

It is important to consider that, when cells are exposed continuously to nanoparticles as in Figure 1E, the measured cell fluorescence is the result of the combination of uptake and competing “exit” processes. These include the dilution of the internalized nanoparticles by cell division [38,39], eventual nanoparticle export or degradation and also the release and exit of the fluorescent drug load. While export of nanoparticles by cells in most cases has not been observed [38,40,41], for degradable nanomaterials, the loaded cargo (drugs or fluorescent labels, such as in our case SRB) can be released into the surrounding environment [42]. Thus, different methods need to be applied to distinguish all these different contributions to the uptake kinetics.

**Figure 2:**
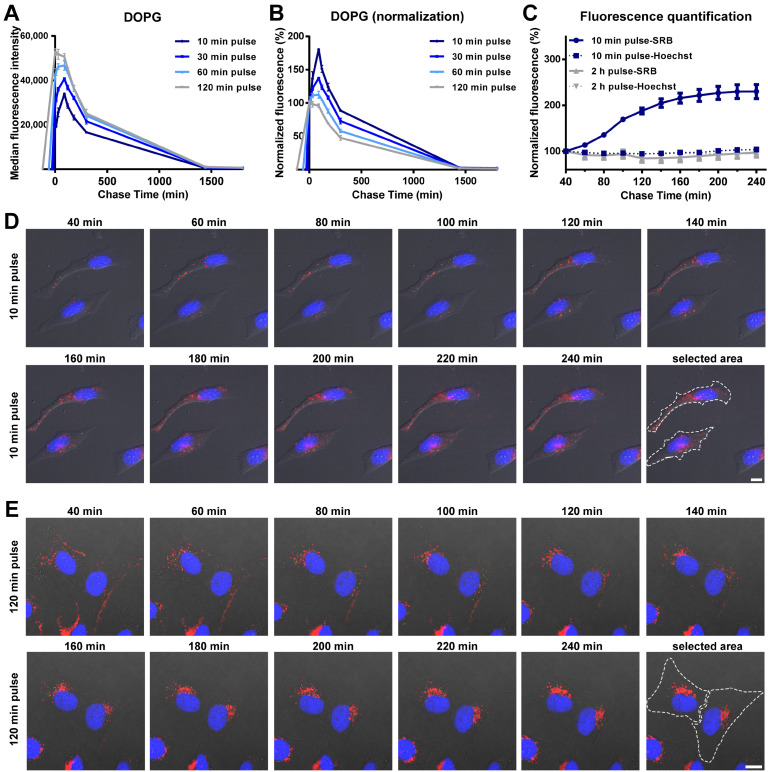
Intracellular release behavior of SRB from DOPG liposomes. (**A**,**B**) Release kinetics in HeLa cells after exposure to DOPG liposome for different times. Briefly, HeLa cells were exposed to 62 µg/mL DOPG liposomes in cMEM for 10, 30, 60 and 120 min (pulse), prior to 3 washes with cMEM to remove the excess liposomes and further incubation in fresh cMEM without liposomes (chase). Cells were then collected at different chase times for flow cytometry analysis as described in Methods. The results are the average and standard deviation over 2 replicates of the median cell fluorescence intensity obtained by flow cytometry. In (**B**), the same data are shown after normalization for the fluorescence at 0 h chase. (**C**) SRB and Hoechst fluorescence quantification from fluorescence imaging of live HeLa cells after 10 min or 120 min exposure to 62 µg/mL DOPG liposomes (pulse) and chase in fresh cMEM without liposomes. The mean fluorescence intensity of two separate cells in the imaging field (see Figure 2D–E for area selection) was calculated and normalized for the starting fluorescence at the beginning of the imaging. The results are the average and standard deviation of the results obtained over 2 cells. The solid lines show the results for SRB and the dash lines for the Hoechst to stain the nuclei. (**D**,**E**) Fluorescence images of HeLa cells after exposure to 62 µg/mL DOPG liposomes (pulse) for 10 min (**D**) and 120 min (**E**) and chase in fresh cMEM without liposomes for up to 240 min. Fluorescence images were taken every 20 min, starting 40 min after liposomes were removed (40 min chase). Blue: Hoechst stained nuclei. Red: SRB encapsulated in liposomes or free SRB. Scale bar: 10 µm.

In order to determine the drug release profiles of liposomes inside cells, we have used so-called “pulse and chase experiments”, in which cells are exposed to liposomes for different times (pulse), followed by removal of the extracellular liposome source in order to monitor the intracellular load over time (chase), excluding uptake. For DOPG liposomes, an initial increase in cell fluorescence intensity was detected during the chase, followed by a progressive decrease in the average fluorescence (Figure 2A and the same after normalization for the cell fluorescence at 0 min chase in Figure 2B). Interestingly, by changing the pulse time between 10 and 120 min, we found that this initial cell fluorescence increase was higher the shorter the pulse time (Figure 2B). In all cases the highest intensity was reached roughly 120 min after uptake started, and the effect was not detected after a 120 min pulse. These results were further confirmed by live fluorescence imaging (Figure 2C–E and corresponding Videos S1 and S2). As shown in Figure 2D, for cells exposed to DOPG liposomes for 10 min, the brightness of vesicles containing SRB increased during the chase. In contrast, after 120 min pulse, no increase in the SRB signal was observed (Figure 2C,E). The nuclear fluorescence of Hoechst was also quantified and remained stable during the whole chase period (also in Figure 2C), suggesting that the effect was not simply due to a change in the focal plane during acquisition.

**Figure 3:**
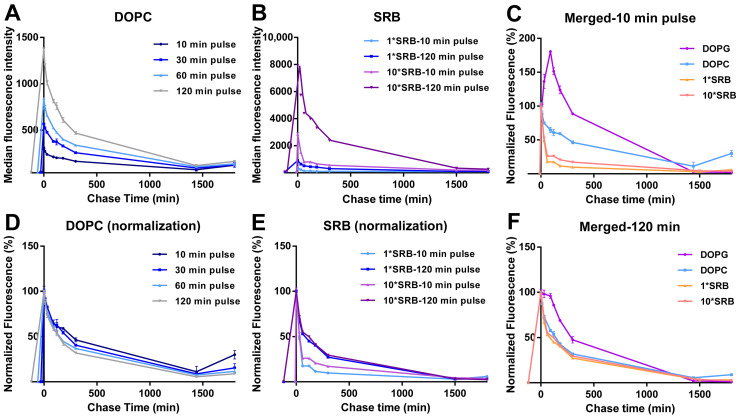
Intracellular release behavior of DOPC liposomes and free SRB. Release kinetics in HeLa cells after exposure to (**A**,**D**) 62 µg/mL DOPC liposomes in cMEM for 10, 30, 60 and 120 min (pulse) or (**B**,**E**) 5 µM SRB (1*SRB) or 50 µM (10*SRB), followed by removal of the extracellular liposome dispersion and further incubation in fresh cMEM without liposomes (chase). The length of the pulse in each experiment is indicated on the x axis as a negative time point (prior to the start of the chase). Cells were then collected at different chase times for flow cytometry analysis as described in Methods. The results are the average and standard deviation over 2 replicates of the median cell fluorescence intensity obtained by flow cytometry. In (**D**,**E**), the same data are shown after normalization for the fluorescence at 0 h case. In (**C**,**F**), the normalized release kinetics after 10 min pulse (**C**) or 120 min pulse (**F**) are overlapped for comparison, including the results for DOPG liposomes (from Figure 2B).

The DOPC liposomes showed very different intracellular drug release behavior. In this case, for all pulse times, uptake was followed by a gradual decrease in cell fluorescence during the whole chase period, with comparable kinetics (Figure 3A and the same after normalization for the cell fluorescence at 0 min chase in Figure 3D). 

Overall, these results suggested that the increase in fluorescence observed for DOPG liposomes during the chase was due to the release and de-quenching of the encapsulated SRB (as shown in Figure 1A for liposomes in 1% triton). The different position of the peak after the different pulse times suggests that this occurred roughly 120 min after the first liposomes were internalized. The loss of this effect after a 120 min pulse, for which no increase in fluorescence is observed during the chase, suggested that other factors may contribute to the release kinetics, which become more visible around that time scale. For instance, eventual effects on liposome stability after interaction with serum and corona formation may become more visible at increasing pulse length, thus with the aging of the liposome dispersions (as we discuss later in Section 3.3). Similarly, cell division is known to lead to a decrease in the internalized nanoparticle load, thus the effects of cell division on the average cell fluorescence become more visible at longer pulse times (and during chase).

In order to gain more information on these results, similar experiments were performed for cells exposed to the same amount of free SRB and, because of its low uptake efficiency, 10 times higher SRB concentration (Figure 3B,E). The results showed a fast cell fluorescence decay at both concentrations, slightly faster for the shorter pulse time. This confirmed that free SRB can be exported from cells. The different exit rate may suggest that, with longer exposure, SRB is trafficked deeper inside cells, thus it has to cross more barriers before is released outside cells. A direct comparison of the different chase kinetics for liposomes and SRB (Figure 3C,F) facilitates seeing that, after a 10 min pulse (Figure 3C), while SRB exited cells very rapidly, the fluorescence decay was slower for both liposomes, possibly because of the time required for SRB first to release from liposomes and then—once free—to exit from cells. The fact that only with DOPG was this peculiar burst and increase in fluorescence observed during the chase suggested that the release of SRB may be very fast for the DOPG liposomes and instead more gradual over time for DOPC. However, we cannot fully exclude that a similar burst release may not be detectable in the case of DOPC liposomes due their much lower uptake efficiency. Additionally, we previously showed that the two liposomes are internalized by cells using different mechanisms [36]. Whether differences in uptake mechanisms may also contribute to the observed differences in intracellular release kinetics remains to be determined. The fact that after a 120 min pulse instead (Figure 3F), the DOPG burst was not visible anymore and all kinetic curves became comparable, possibly indicates that, after longer exposure, when both free SRB and liposomes have been trafficked deeper inside cells, both free SRB and the SRB released from the liposomes have to cross more barriers to exit cells.

**Figure 4:**
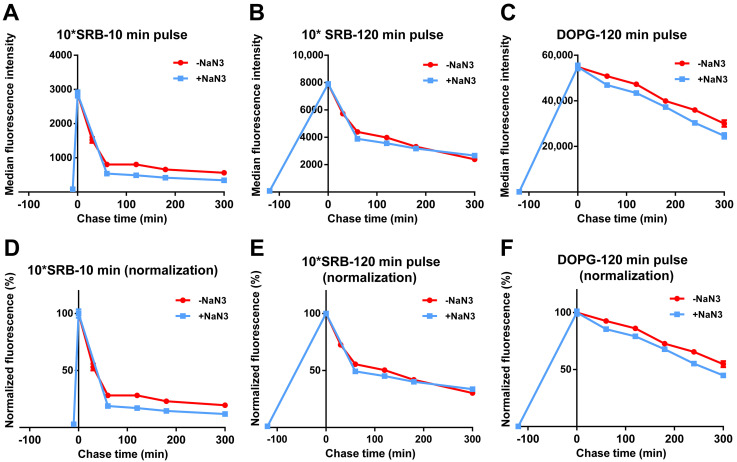
Release of free SRB and DOPG liposomes in energy-depleted cells. HeLa cells were exposed to (**A**,**B**,**D**,**E**) 50 µM SRB (10*SRB) or (**C**,**F**) 62 µg/mL DOPG liposomes in cMEM for (**A**,**D**) 10 min, or (**B**,**C**,**E**,**F**) 120 min (pulse), followed by removal of the extracellular liposome dispersion and further incubation in fresh cMEM without liposomes (chase) in cMEM without liposomes in standard conditions (−NaN_3_) or in the presence of 5 mg/mL NaN_3_ (+NaN_3_) to deplete cell energy. The length of the pulse in each experiment is indicated on the x axis as a negative time point (prior to the start of the chase). Cells were then collected at different chase times for flow cytometry analysis as described in Methods. The results are the average and standard deviation over 2 replicates of the median cell fluorescence intensity obtained by flow cytometry. In (**D**–**F**), the same data are shown after normalization for the fluorescence at 0 h chase. The data obtained from cells exposed to SRB in cMEM in standard condition (−NaN_3_) are reproduced from Figure 3.

We then tested whether the release of SRB or liposomes was energy-dependent. For this, cells were exposed to free SRB or DOPG, followed by similar chase experiments but in the presence of NaN_3_. As shown in Figure 4 and Figure S2, the fluorescence decay after a pulse of free SRB was not reduced by NaN_3_, suggesting that free SRB exits cells via passive mechanisms. As previously observed (Figure 2 and Figure 3), the decay for DOPG was slower, likely because SRB must first be released from liposomes and then can exit cells. Interestingly, the fact that also for DOPG the decay is not affected by energy depletion with NaN_3_ suggested the absence of active liposome export. Thus, after the active uptake of SRB encapsulated in the liposomes, SRB is released from the liposomes inside cells and only once free can exit cells with kinetics comparable to what is observed for free SRB.

### 3.3. Stability of Liposomes in Biological Environments

**Figure 5:**
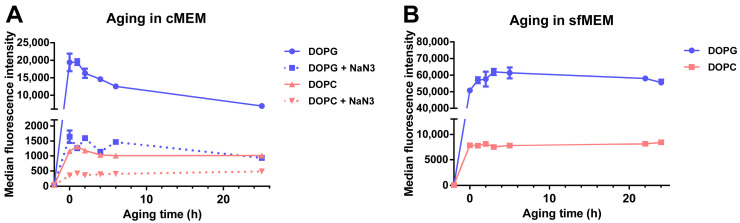
Cellular uptake of liposomes after aging in cMEM or serum free medium (sfMEM). (**A**,**B**) Cellular uptake of DOPG and DOPC liposomes in cMEM in the presence or absence of NaN_3_ (**A**) and liposomes in sfMEM (**B**). Briefly, 62 µg/mL DOPG or DOPC liposomes in cMEM (**A**) or sfMEM (**B**) were maintained in cell culture conditions in a humidified atmosphere with 5% CO_2_ at 37 °C for increasing times (aging). Then after different aging times, the dispersion was collected and added to cells for 2 h, prior to quantification of cell fluorescence by flow cytometry. For liposomes in cMEM, uptake experiments were performed in the presence or absence of 5 mg/mL NaN_3_ (see Methods for details). The results are the average and standard deviation over 2 replicates of the median cell fluorescence obtained by flow cytometry.

Another important factor that affects drug release from liposomes and consecutively also similar uptake studies in cells is the stability of liposomes in biological environment, following the adsorption of proteins on their surface and corona formation [43,44]. In order to determine stability in a biological environment, liposomes were incubated in cMEM under cell culture conditions for increasing times, then the “aged” dispersions were exposed to cells for 2 h, and the uptake was measured by flow cytometry (Figure 5A). The results showed that, for DOPG liposomes, the cell fluorescence decreased substantially for cells exposed to dispersions of increasing aging time (roughly 35% and 64% fluorescence reduction with dispersions aged 6 h and 25 h in cMEM, respectively). The effect was smaller for DOPC liposomes, for which 11% and 44% cell fluorescence reduction was observed with dispersions aged for 6 h and 25 h, respectively. Interestingly, instead, no decrease was observed when the same experiment was performed with liposome dispersions maintained in serum free medium (sfMEM) in cell culture conditions (Figure 5B). Overall, these results suggested that the interactions with biological medium supplemented with serum partly affected the bilayer stability. We previously showed that the negatively charged DOPG liposomes adsorb a higher amount of proteins in their corona in comparison to the zwitterionic DOPC liposomes [32], and this may explain the stronger effects observed for DOPG. Additional effects due to specific differences in corona composition may also contribute to these differences. 

It is important to note that characterization in cell culture medium supplemented with serum showed that both liposome dispersions remained stable after 24 h incubation in biological conditions (5% CO_2_ at 37 °C) [32]. The same conditions were applied here for aging the dispersions. Therefore, we can exclude the possibility that the observed decrease in cell fluorescence with aged dispersions is due to aggregation, but rather it is due to the partial release of SRB from the liposomes upon interaction with serum and corona formation, with stronger effects in the case of DOPG because of its higher protein adsorption. This is in agreement with similar studies in literature. For example. Allen et al. reported that the presence of serum significantly increased the leakage of small unilamellar liposomes with various compositions [45], and Hernfindez-Caselles et al. showed that liposomes with neutral charge containing phosphatidycholine were the most stable, while those containing negatively charged phospholipids were very unstable [46].

To further confirm this, size-exclusion chromatography (SEC) was used to separate released SRB from the liposomes (Figure S3) [24], taking advantage of their very different size. SEC showed that liposomes stored in PBS were stable with no residual free SRB or signs of SRB leakage. However, once incubated in cMEM in cell culture conditions (5% CO_2_, 37 °C and a humidified atmosphere), a fraction of free SRB could be detected, which increased with increasing aging time. For DOPG liposomes, the free SRB separated from SEC corresponded to up to 85% of all SRB after 24 h aging in medium with serum. Instead, in line with the smaller decrease in cell fluorescence upon exposure to aged liposomes (Figure 5A), the effect was much smaller for DOPC liposomes, confirming their higher stability. It is important to specify that the degree of leakage observed with SEC after liposome aging is likely to be much higher than during exposure to cells, due to the interactions between lipids and polymer beads in the column, known to accelerate liposome leakage [47]. Reynolds et al. suggested a step of gel pre-saturation with lipids to avoid liposome loss during SEC [48]; however, this could introduce contaminations [47], further complicating the results. An aging experiment, followed by cellular uptake as shown in Figure 5, can be used as a more straightforward alternative for testing similar effects due to the exposure of liposomes to serum. 

Overall, even though the fluorescence decrease observed in cells exposed to aged DOPG dispersions was rather strong (Figure 5A), the uptake of free SRB was much lower (Figure S1) (around 2k A.U. after 24 h exposure to free SRB, as opposed to around 10k A.U. after only 2 h exposure to DOPG aged for 24 h). Additionally, as shown in Figure 5A, also for the aged liposomes, exposure to NaN_3_ strongly decrease uptake (87% and 55% reduction for DOPG and DOPC liposomes, respectively, after 24 h aging, as opposed to around max 30% for free SRB). The much lower uptake efficiency of free SRB and the strong effect of sodium azide on the uptake of aged liposomes, together, confirmed that, despite the leakage upon interaction with serum, the fluorescence detected in cells exposed to aged liposomes came primarily from the active uptake of the residual SRB loaded inside the liposomes rather than the uptake of the leaked SRB.

**Figure 6:**
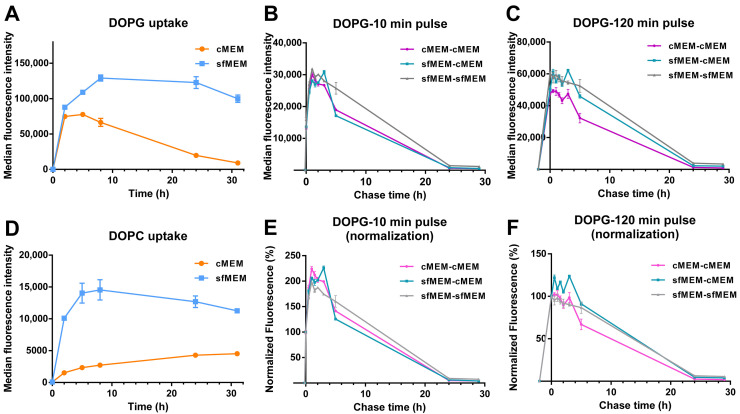
Comparison of liposome uptake and release in cMEM and sfMEM. (**A**,**D**) Uptake kinetics of DOPG liposome (**A**) and DOPC liposome (**B**) in cMEM and sfMEM. HeLa cells were exposed to 62 µg/mL DOPG or DOPC liposomes dispersed in cMEM or sfMEM and collected after different exposure times for flow cytometry measurement as described in Methods. The results are the average and standard deviation over 2 replicates of the median cell fluorescence intensity obtained by flow cytometry. (**B**,**C**,**E**,**F**) Release kinetics of HeLa cells after exposure to DOPG liposomes dispersed in different media. HeLa cells were exposed to 62 µg/mL DOPG liposomes in cMEM or sfMEM for (**B**,**E**) 10 min or (**C**,**F**) 120 min (pulse), followed by removal of the extracellular liposome dispersion and further incubation (chase) in cMEM or sfMEM without liposomes. The length of the pulse in each experiment is indicated on the x axis as a negative time point (prior to the start of the chase). Cells were then collected at different chase times for flow cytometry analysis as described in Methods. The results are the average and standard deviation over 2 replicates of the median cell fluorescence intensity obtained by flow cytometry. In (**E**,**F**), the same data are shown after normalization for the fluorescence at 0 h chase.

Finally, given that the data of Figure 5 suggested that interaction with serum and corona formation strongly affected liposome stability, in particular for DOPG, as an ulterior proof of the impact of serum interactions on liposome stability, uptake studies were performed in artificial serum free conditions (Figure 6A,D). In this case, uptake levels were overall higher, as often reported for bare particles in the absence of serum [49,50]. Interestingly, for both liposomes, the uptake kinetics were very different than in serum (Figure 1E) and showed a linear increase followed by a plateau. Thus, in absence of serum the liposome bilayers remained stable (as shown by the aging experiments of Figure 5B) and because of this and because of the overall higher uptake efficiency in these conditions, the peculiar decrease in the uptake kinetics observed for DOPG in complete medium with serum (Figure 1E) was not observed. On the contrary, pulse and chase experiments showed that as observed in medium with serum (Figure 2), after 10 min pulse, an increase in cell fluorescence could be detected also for DOPG liposomes added to cells in serum free medium, and this increase was not observed after 120 min pulse (Figure 6B,C,E,F). Thus, also in serum free conditions, a burst of fluorescence was observed inside cells due to the de-quenching of the encapsulated SRB upon release.

## 4. Conclusions

Optimal liposomal formulations should retain their drug load in blood and as they distribute in the body and then release it once at their target. Very stable formulations could ensure no drug is lost during delivery but may show limited release at the target. Thus, liposome stability and release properties can be contrasting and need to be tuned in order to optimize formulations with the required delivery and release properties. 

Classic in vitro release studies with methods such as dialysis or centrifugation may be hard to apply to complex biological environments, and methods to determine liposome stability in complex biological media and intracellular release kinetics are highly sought. Herein, we showed that the aging of liposome dispersions and size-exclusion chromatography, combined with uptake kinetics and pulse and chase experiments by flow cytometry, can be used for this purpose. 

The methods presented allow addressing at least in part some of the limits of simpler release studies, in order to characterize liposome stability in complex biological fluids, as well as to determine release kinetics inside cells. Similar approaches can also be used for nanomedicine formulations other than liposomes.

Thus, the stability and release properties of DOPG and DOPC liposomes encapsulating comparable amounts of SRB were compared. The DOPG liposomes were able to deliver very high amounts of SRB in short time inside cells; however, the high uptake efficiency and fast release properties inside cells were also accompanied by a substantial loss of the SRB load outside cells upon interaction with serum and corona formation. On the contrary, DOPC uptake efficiency was much lower and the release more gradual and sustained over time, and this formulation also showed higher stability in biological conditions when exposed to serum. Depending on the application and drug load, liposome formulations and other nanomedicines can be tuned to achieve the required balance between stability in serum and drug release at the target.

## Data Availability

The data presented in this study are available on request from the corresponding author.

## Supplementary Materials

The following supporting information can be downloaded at: https://www.mdpi.com/article/10.3390/biom13010059/s1, Figure S1: uptake of sulforhodamine B (SRB) in by Hela cells; Figure S2: Release of free SRB in energy-depleted cells; Figure S3: Size-exclusion chromatography (SEC) of liposomes after aging in cMEM; Video S1: Time-lapse movie of HeLa cells after exposure to 62 µg/mL DOPG liposomes for 10 min (pulse) and chase in fresh cMEM without liposomes for up to 240 min; Video S2: Time-lapse movie of HeLa cells after exposure to 62 µg/mL DOPG liposomes for 120 min (pulse) and chase in fresh cMEM without liposomes for up to 240 min.
