# Supplementary material for: Tuning Liposome Stability in Biological Environments and Intracellular Drug Release Kinetics
Source: Biomolecules. 2022 Dec 27;13(1):59. doi: 10.3390/biom13010059 (PMC9855369; doi:10.3390/biom13010059)
Supplement: Supplementary file 1 [file biomolecules-13-00059-s001.zip › biomolecules-2080057-final-supplementary/Yang et al. SI-Biomolecules-proofs.pdf]

Supplementary Materials

Tuning Liposome Stability in Biological Environments and Intracellular Drug Release Kinetics

Keni Yang [1,†], Karolina Tran [1,#] and Anna Salvati [1,*]

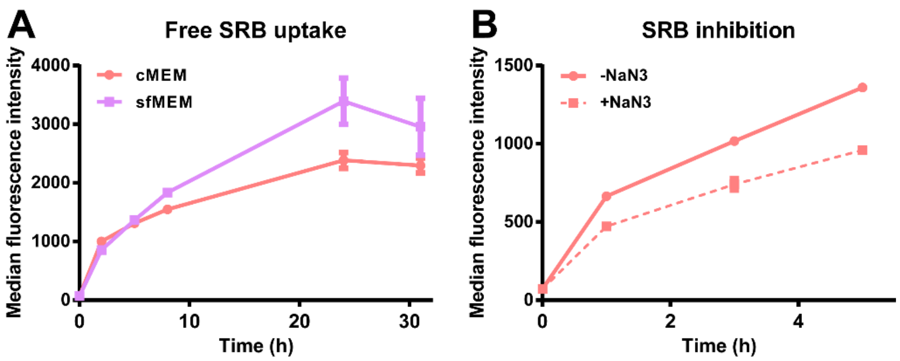

Figure S1. Uptake of sulforhodamine B (SRB) in by Hela cells. (A) Uptake kinetics of free SRB in cMEM or serum free medium (sfMEM). HeLa cells were exposed to 5 μM SRB dispersed in cMEM or sfMEM, and after different exposure times cells were collected for flow cytometry measurement as described in Methods. The results are the average and standard deviation over 2 replicates of the median cell fluorescence intensity obtained by flow cytometry. (B) Uptake of free SRB in energy depleted cells. HeLa cells were pretreated with 5 mg/ml sodium azide (NaN_3) for 30 min and exposed to 5 μM SRB dispersed in cMEM in the presence of NaN_3 (+NaN_3). Uptake in standard conditions was also measured for comparison (-NaN_3). Cells were then collected at different time points for flow cytometry measurement as described in Methods. The results showed around maximum 30% uptake inhibition of free SRB in the presence of NaN_3. In the cells exposed to SRB in the presence of NaN_3 at 3 h and 5 h, double peaks in the cell fluorescence distribution were observed from flow cytometry, with one portion of cells containing higher signal than cells exposed to SRB in standard condition. Also, only 10000 cells were collected for flow cytometry analysis after cells exposed in NaN_3 condition for 5 h. These might be because of the toxic effect of NaN_3 leading to cells dying, thus showing compromised (higher) cell membrane permeability.

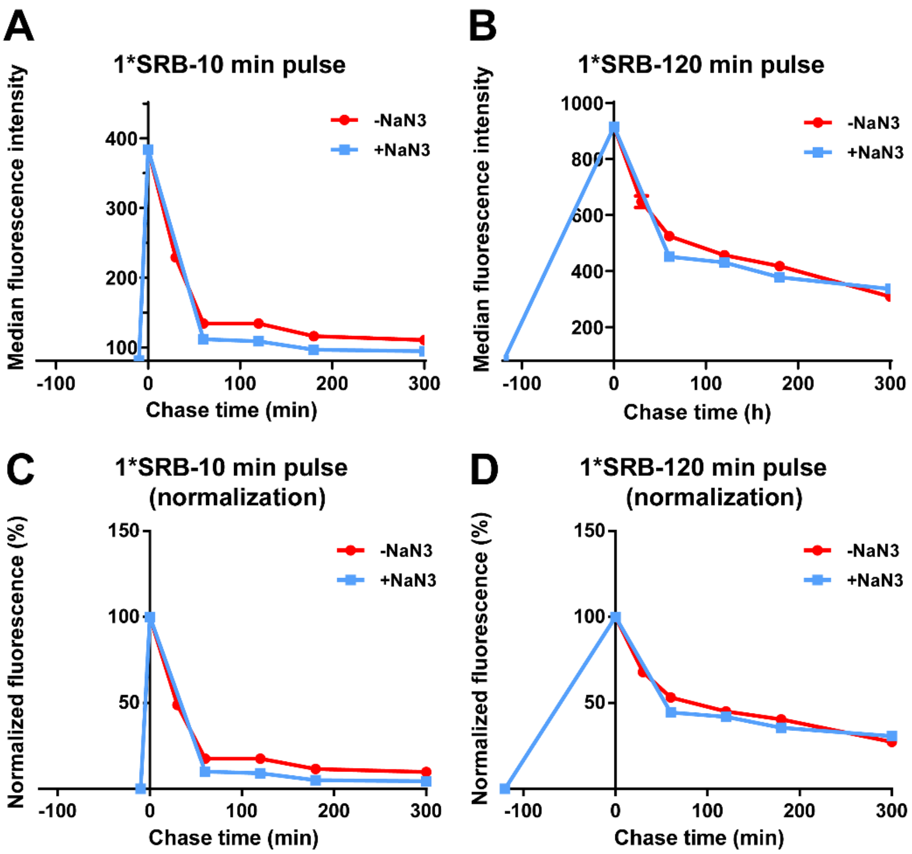

Figure S2. Release of free SRB in energy-depleted cells. HeLa cells were exposed to 5 µM SRB (1*SRB) for (A and C) 10 min, or (B and D) 120 min (pulse), followed by removal of the extracellular liposome dispersion and further incubation (chase) in cMEM without liposomes in standard conditions (- NaN_3) or in the presence of 5 mg/ml NaN_3 (+ NaN_3) to deplete cell energy. The length of the pulse in each experiment is indicated on the x axis as a negative time point (prior to the start of the chase). Cells were then collected at different chase times for flow cytometry analysis as described in Methods. The results are the average and standard deviation over 2 replicates of the median cell fluorescence intensity obtained by flow cytometry. In (B) and (D) the same data are shown after normalization for the fluorescence at 0 h chase. The data obtained from cells exposed to SRB in cMEM in standard condition (- NaN_3) are reproduced from Fig. 3. The results showed that the fluorescence decay in cells after a pulse of free SRB cannot be inhibited by NaN_3, indicating SRB is released from cells by passive mechanisms.

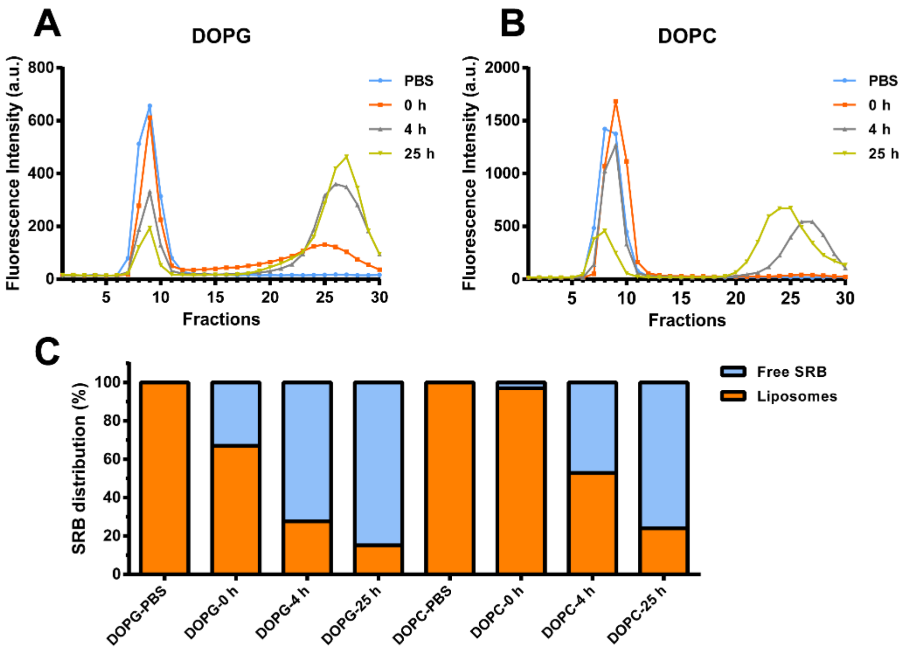

Figure S3. Size exclusion chromatography (SEC) of liposomes after aging in cMEM. (A-B) Elution profile of DOPG (A) and DOPC (B) liposomes in PBS and after aging of the dispersion by incubation in cMEM. Briefly, 1 ml 62 µg/ml liposomes were dispersed in PBS or in cMEM (without phenol red) in Eppendorf tubes with closed caps, followed by incubation in cell culture conditions (37 ºC, 5% CO_2 and humidified atmosphere) for 0, 4 and 25 h (aging). The dispersions were then loaded into a Sepharose CL-4B column. Elutes were collected (0.5 ml per fraction) and 50 µl of each fraction was mixed with 50 µl 1% triton (v/v) followed by fluorescence measurement (see Methods for details). We note that the fluorescence intensity of the fractions without liposome or free dye was close to background, suggesting that potential interference of triton on the fluorescence measurement was negligible. (C) Quantification of free SRB and SRB encapsulated in liposomes from elution profiles. From the elution profiles of panels A and B, the areas of the peaks corresponding to SRB encapsulated in liposome (roughly fractions 6-11) and free SRB (roughly fractions 18-30) were calculated using Area Under Curve function from GraphPad Prism software and used to estimate the fraction of encapsulated and free SRB in each sample. The results showed that exposure to FBS and corona formation led to release of SRB, with stronger effects for DOPG liposomes. However, it is likely that the effects are amplified by the interactions with the gel in the column (see manuscript for details).

Video S1. Time-lapse movie of HeLa cells after exposure to 62 μg/ml DOPG liposomes for 10 min (pulse) and chase in fresh cMEM without liposomes for up to 240 min. Fluorescence images were taken every 20 min, starting 40 min after liposome were removed (40 min chase). Blue: Hoechst stained nuclei. Red: SRB encapsulated liposomes or free SRB. Scale bar: 10 μm. Images extracted from this video are shown in Figure 2D.

Video S2. Time-lapse movie of HeLa cells after exposure to 62 μg/ml DOPG liposomes for 120 min (pulse) and chase in fresh cMEM without liposomes for up to 240 min. Fluorescence images were taken every 20 min, starting 40 min after liposome were removed (40 min chase).Blue: Hoechst stained nuclei. Red: SRB encapsulated liposomes or free SRB. Scale bar: 10 μm. Images extracted from this video are shown in Figure 2E.
